# Supplementary material for: Insights into the aetiology of snoring from observational and genetic investigations in the UK Biobank
Source: Nat Commun. 2020 Feb 14;11:817. doi: 10.1038/s41467-020-14625-1 (PMC7021827; doi:10.1038/s41467-020-14625-1)
Supplement: Supplementary file 4 — Supplementary Information [file 41467_2020_14625_MOESM4_ESM.pdf]

Insights into the aetiology of snoring from observational and genetic investigations in the UK Biobank

Campos and García-Marín et al.

Contents

SUPPLEMENTARY FIGURES AND TABLES 2

Supplementary Figure 1. GWAS and sensitivity GWAS results. 2

Supplementary Figure 2. MAGMA Gene-based test association analyses 3

Supplementary Figure 3. MAGMA Tissue Enrichment Analysis. 4

Supplementary Figure 5. Cross-sex effects size of snoring associated SNPs. 7

Supplementary Figure 6. Genetic scoring results.. 8

Supplementary Table 1. Phenotypic correlations of snoring and associated factors............................. 9

Supplementary Table 2. Full MR results.. 10

Supplementary Table 3. Sample sizes for discovery, sex-stratified and sensitivity GWAS analyses.. 14

SUPPLEMENTARY FIGURES AND TABLES

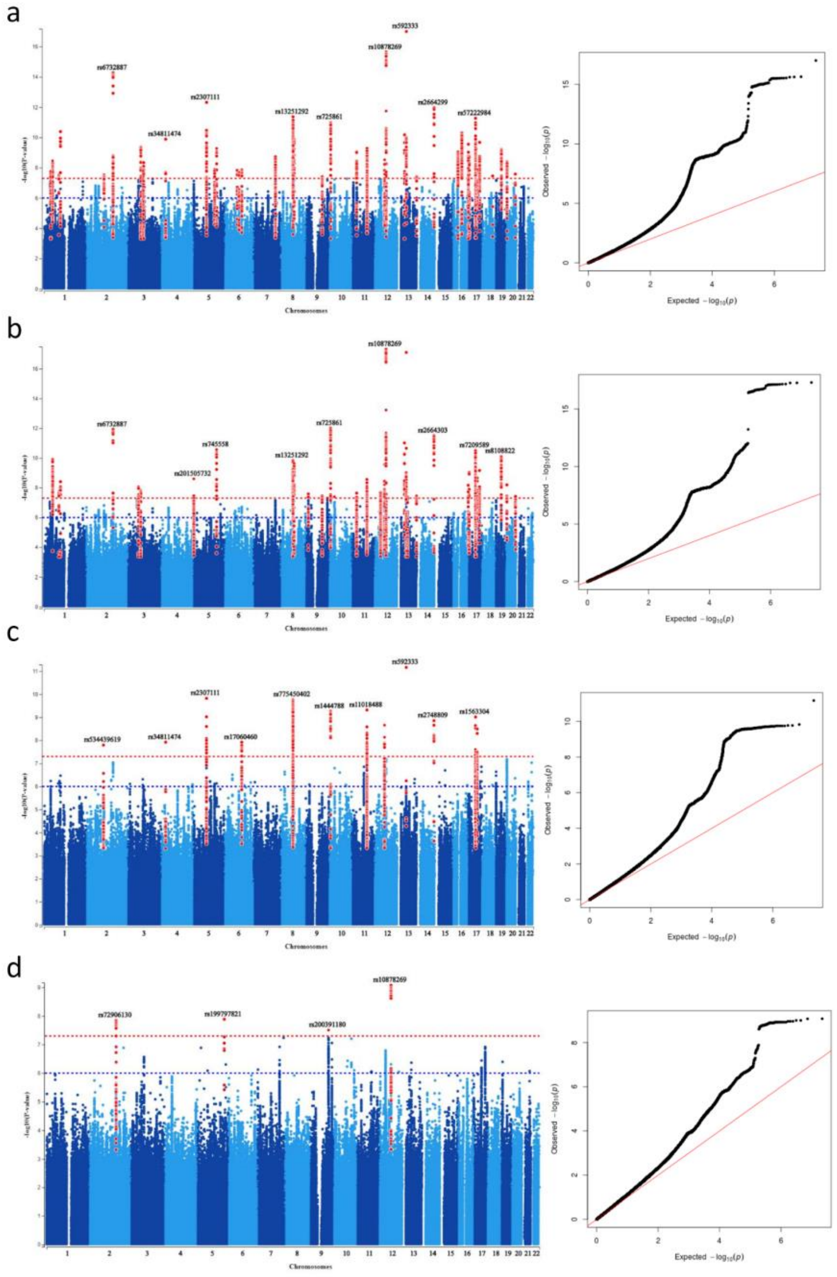

Supplementary Figure 1. GWAS and sensitivity GWAS results.

Supplementary annotated Manhattan plots with LD highlighted and top hits labelled (left panels). quantile-quantile plots (QQplots) are depicted on the right panels. a) Results for snoring GWAS. b) Results for snoring GWAS adjusting for BMI as a covariate. c) Results for snoring GWAS

performed only on the female subset of the sample. d) Results for snoring GWAS performed only on the male subset of the sample.

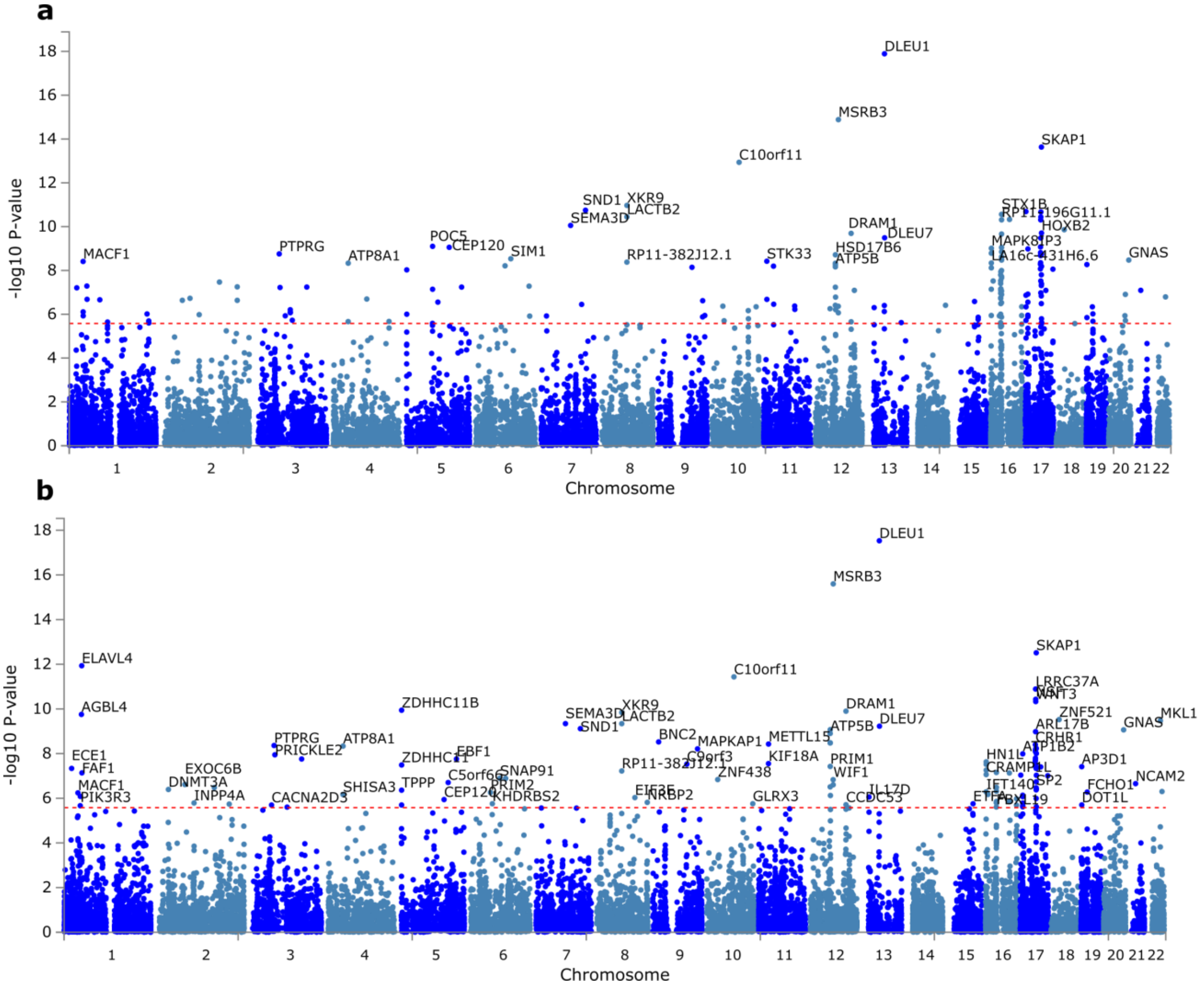

Supplementary Figure 2. MAGMA Gene-based test association analyses

Manhattan plots depicting the gene-based test association analyses performed with MAGMA for snoring (a) and snoring adjusted for BMI (b). Some gene labels were removed or rearranged for better clarity. The red line indicated the bonferroni corrected genome-wide significance threshold (p<2.636e-6; 18,971 tested genes).

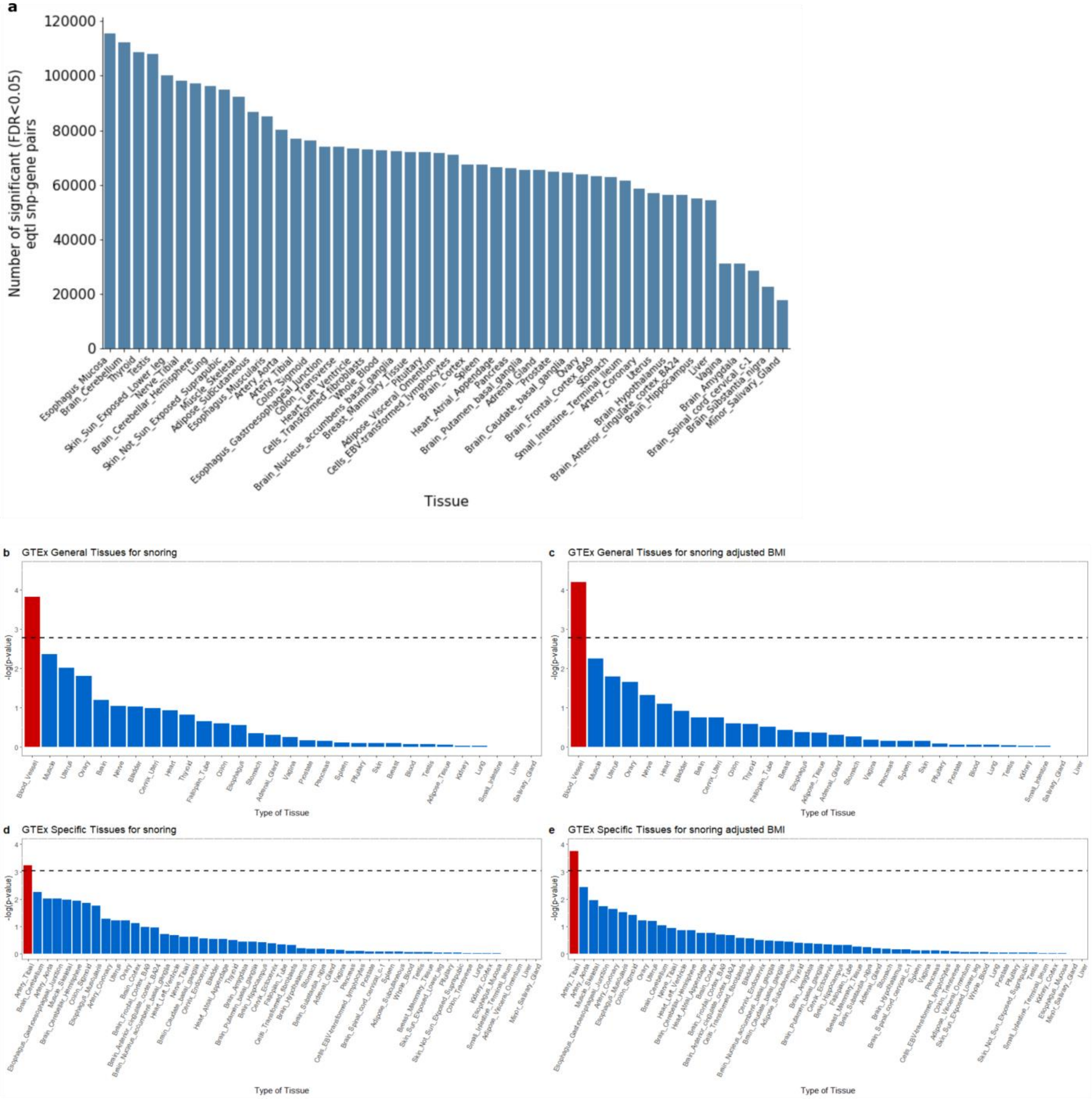

Supplementary Figure 3. MAGMA Tissue Enrichment Analysis.
Bar plots showing gene set enrichment analyses for eQTL mapping (a) and tissue-specific gene expression based on the GWAS for snoring (a,b,d) and snoring adjusted for BMI (c,e). Analyses performed with MAGMA. Red bars indicate significantly enriched tissues (i.e. crossing the bonferroni corrected significance threshold shown with a dashed line).

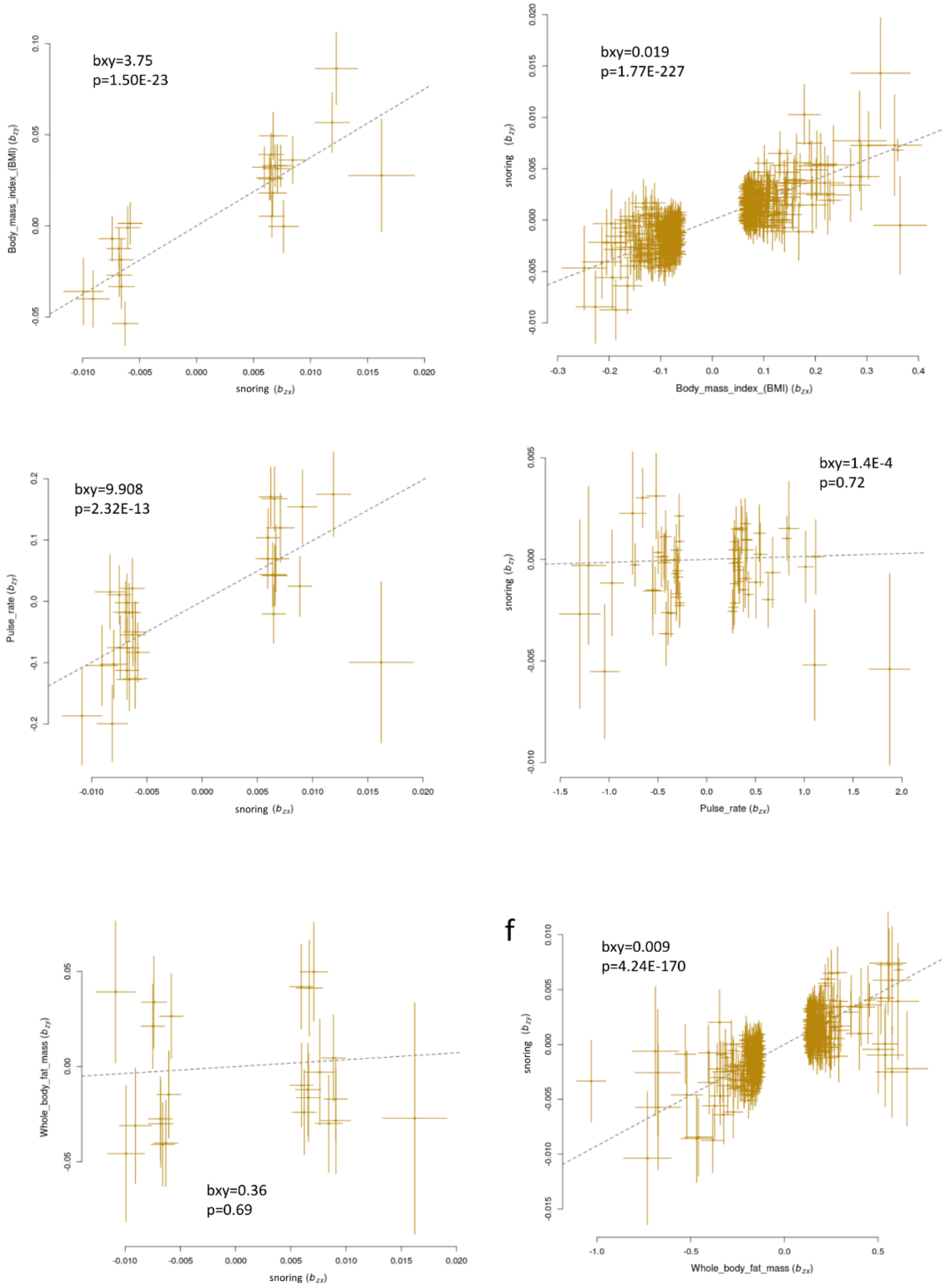

bxy=3.75
p=1.50E-23
Body_mass_index_(BMI) (b_{zy})
snoring (b_{zx})
bxy=0.019
p=1.77E-227
snoring (b_{zy})
Body_mass_index_(BMI) (b_{zx})
bxy=9.908
p=2.32E-13
Pulse_rate (b_{zy})
snoring (b_{zx})
bxy=1.4E-4
p=0.72
snoring (b_{zy})
Pulse_rate (b_{zx})
Whole_body_fat_mass (b_{zy})
bxy=0.36
p=0.69
snoring (b_{zx})
f
bxy=0.009
p=4.24E-170
snoring (b_{zy})
Whole_body_fat_mass (b_{zx})

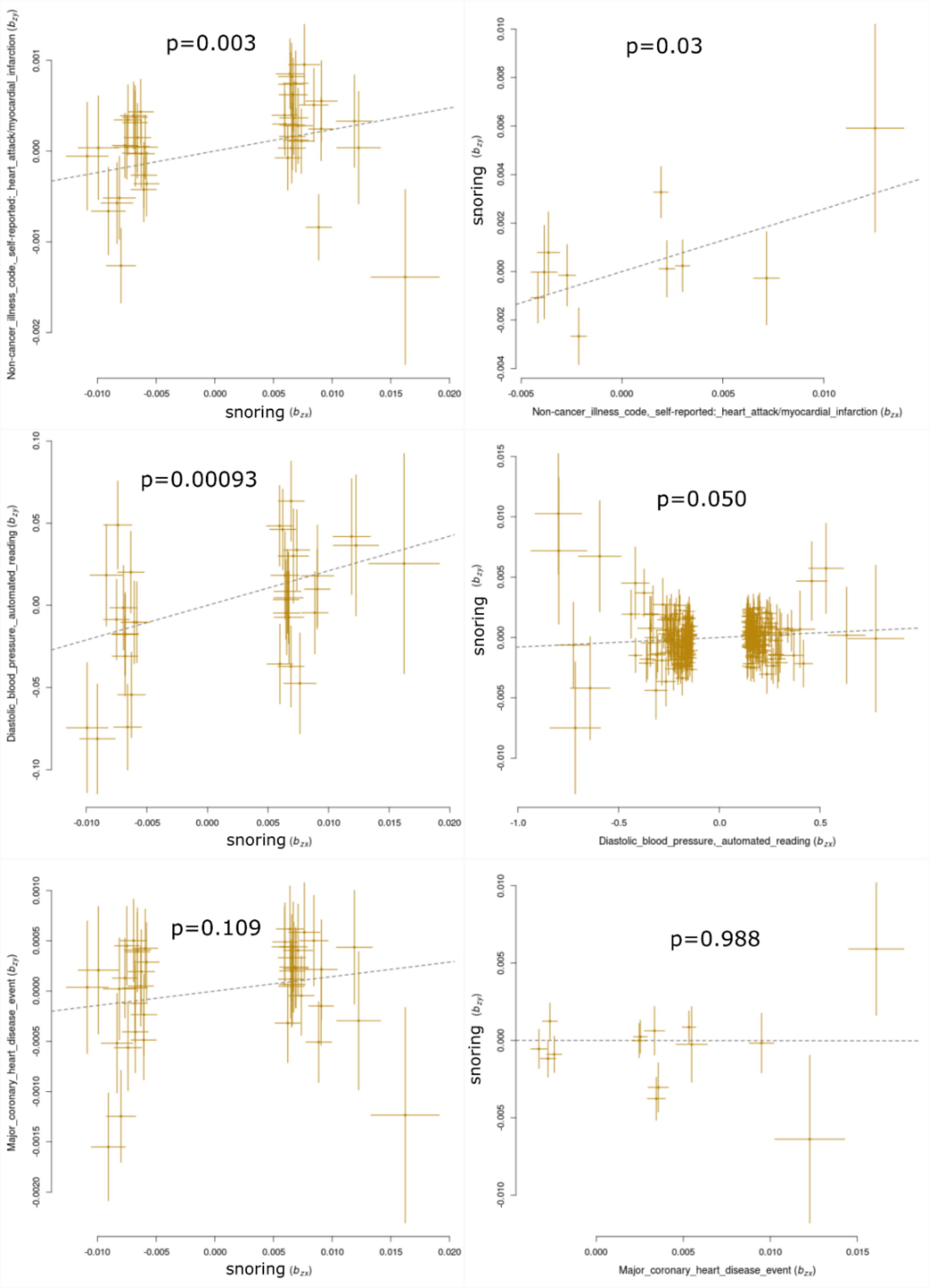

Supplementary Figure 4. Mendelian randomisation effect size plots

Scatter plots depicting the relationship between the effect sizes of SNP instruments for exposures (x-axis) and outcomes (y-axis). If the exposure and the outcome have a causal relationship, then the effects of SNPs (instruments) on the exposure should have proportional effects on trait two. Inset of each plot depicts the estimated causal effect (bxy) and its associated p-value. a,c,e) Show the results of snoring as an exposure while b,d and f show the effects of BMI, Pulse rate or Whole body fat mass as exposures. Statistics such as P–values from GSMR are shown within the plot.

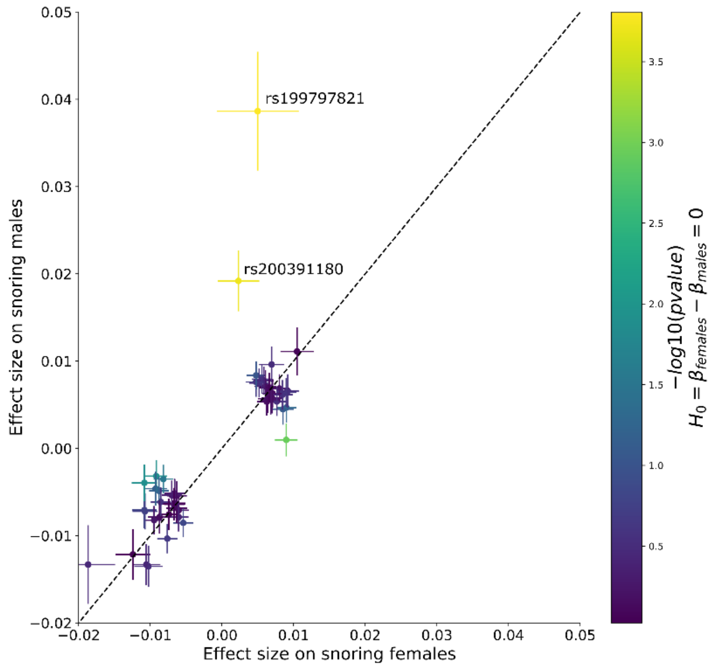

Supplementary Figure 5. Cross-sex effects size of snoring associated SNPs.
Scatter plot showing the effect sizes of main and sex-stratified GWAS independent SNPs in both females (x-axis) and males (y-axis). The colour represents the –log10 p-value of a t-test assessing the difference between effect estimates in males vs. females. Only SNPs with a significant difference after multiple testing correction ($p<0.00098$) have been labeled.

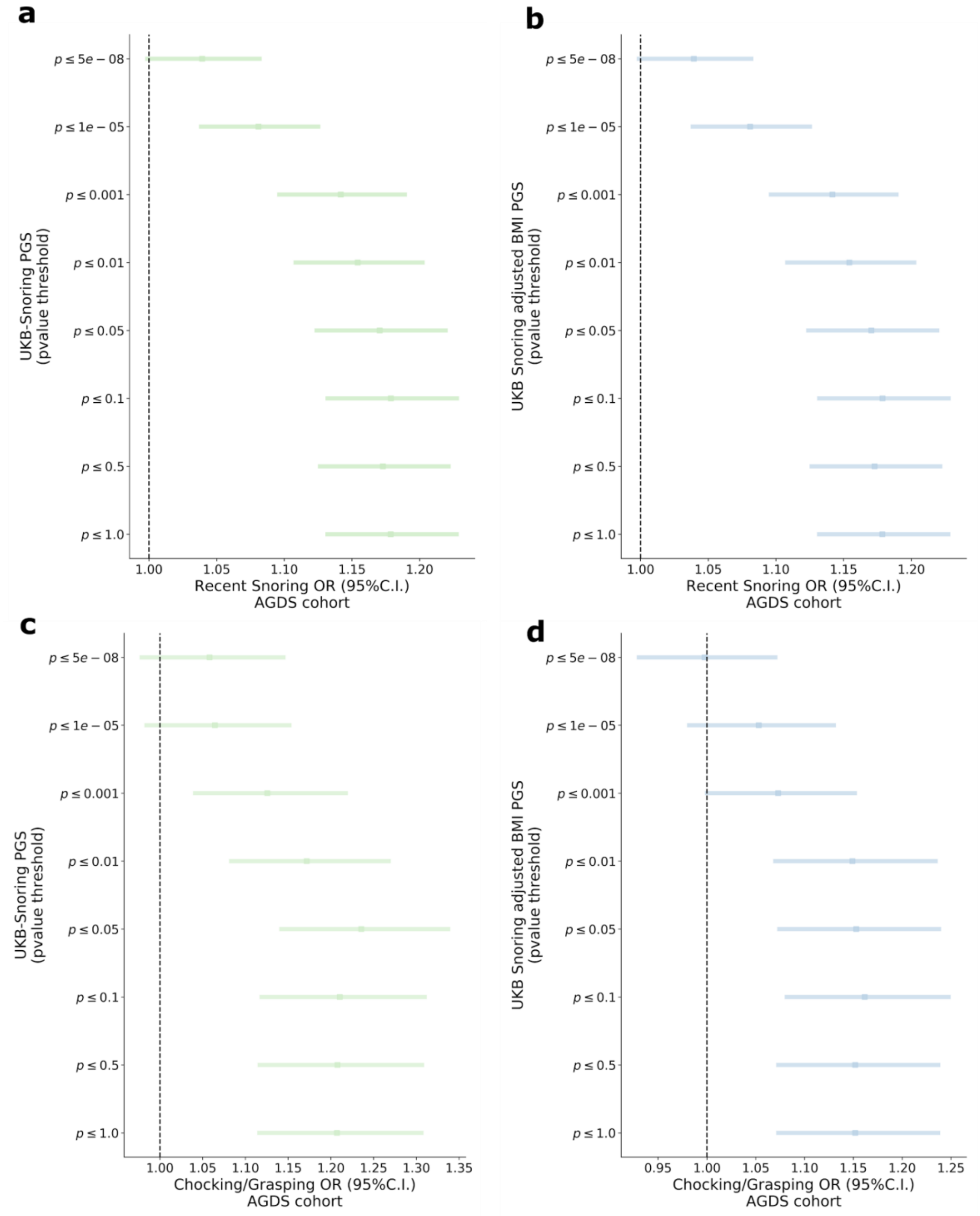

Supplementary Figure 6. Genetic scoring results

Results of association between snoring (a,b) or snoring adjusted for BMI (c,d) and polygenic scores (PGS) for snoring constructed with an increasingly stringent p-value threshold for variant

inclusion (y axis). The x-axis denotes the odds ratio (OR) between the PGS and the phenotype. Bars denote the 95% confidence interval.

Supplementary Table 1. Phenotypic correlations of snoring and associated factors

Predictor	beta (logOR)	S.E.	P-value
Intercept	-4.936	0.09	0.0E+00
Current tobacco [Occasionally]	0.1539	0.04	6.1E-05
Current tobacco [Most Days]	0.3697	0.03	3.3E-41
Alcohol frequency [rarely]	0.1031	0.03	1.5E-04
Alcohol frequency [occasionally]	0.1467	0.03	9.5E-09
Alcohol frequency [frequently]	0.2209	0.02	3.9E-20
Alcohol frequency [very frequently]	0.319	0.02	2.3E-40
Pulse rate	0.003	0	1.0E-07
Sleep_duration	0.0547	0.01	2.0E-21
Whole body fat mass	0.0051	0	4.7E-03
Sex	0.846	0.02	0.0E+00
BMI	0.0891	0	4.0E-136
Age	0.0103	0	1.9E-39
Apnoea	1.1206	0.06	4.5E-80

Supplementary Table 2 Full MR results

Outcome	Exposure	Method	N intruments	b	se	p-val
SnoringMales	BMI_females	MR Egger	140	0.004934	0.004972	0.322802
SnoringMales	BMI_females	Weighted median	140	0.010788	0.002081	2.18E-07
SnoringMales	BMI_females	Inverse variance weighted	140	0.010257	0.001652	5.27E-10
SnoringMales	BMI_females	Weighted mode	140	0.018019	0.010841	0.098745
SnoringMales	BloodPresure_females	MR Egger	109	-0.00484	0.003525	0.172872
SnoringMales	BloodPresure_females	Weighted median	109	-0.00162	0.001062	0.127343
SnoringMales	BloodPresure_females	Inverse variance weighted	109	-0.00053	0.000861	0.538703
SnoringMales	BloodPresure_females	Weighted mode	109	-0.00279	0.002827	0.325856
SnoringMales	PulseRate_females	MR Egger	27	0.005114	0.002123	0.023688
SnoringMales	PulseRate_females	Weighted median	27	0.001416	0.000979	0.147867
SnoringMales	PulseRate_females	Inverse variance weighted	27	0.000731	0.000711	0.30414
SnoringMales	PulseRate_females	Weighted mode	27	0.001597	0.001378	0.256873
SnoringMales	WholeBodyFat_females	MR Egger	140	0.002687	0.002525	0.289189
SnoringMales	WholeBodyFat_females	Weighted median	140	0.005782	0.00107	6.54E-08
SnoringMales	WholeBodyFat_females	Inverse variance weighted	140	0.005423	0.000801	1.28E-11
SnoringMales	WholeBodyFat_females	Weighted mode	140	0.009568	0.006478	0.141916
SnoringFemales	BMI_males	MR Egger	122	0.008276	0.005557	0.139057
SnoringFemales	BMI_males	Weighted median	122	0.01503	0.002137	2.01E-12
SnoringFemales	BMI_males	Inverse variance weighted	122	0.013069	0.001824	7.67E-13
SnoringFemales	BMI_males	Weighted mode	122	0.018003	0.003969	1.36E-05
SnoringFemales	BloodPresure_males	MR Egger	43	-0.00225	0.004089	0.5849
SnoringFemales	BloodPresure_males	Weighted median	43	0.000815	0.001345	0.544755

SnoringFemales	BloodPresure_males	Inverse variance weighted	43	-0.00046	0.001014	0.653696
SnoringFemales	BloodPresure_males	Weighted mode	43	0.001994	0.003132	0.527931
SnoringFemales	PulseRate_males	MR Egger	16	-0.00153	0.003573	0.675229
SnoringFemales	PulseRate_males	Weighted median	16	-0.00038	0.000931	0.680024
SnoringFemales	PulseRate_males	Inverse variance weighted	16	-6.8E-05	0.001016	0.946698
SnoringFemales	PulseRate_males	Weighted mode	16	-0.00039	0.001219	0.75177
SnoringFemales	WholeBodyFat_males	MR Egger	109	0.005074	0.003248	0.121221
SnoringFemales	WholeBodyFat_males	Weighted median	109	0.004301	0.001119	0.000121
SnoringFemales	WholeBodyFat_males	Inverse variance weighted	109	0.005439	0.00098	2.9E-08
SnoringFemales	WholeBodyFat_males	Weighted mode	109	0.000857	0.003072	0.780871
SnoringFemales	HeartAttack_males	MR Egger	9	0.340837	0.252299	0.218755
SnoringFemales	HeartAttack_males	Weighted median	9	0.019489	0.113392	0.86354
SnoringFemales	HeartAttack_males	Inverse variance weighted	9	-0.02545	0.087665	0.7716
SnoringFemales	HeartAttack_males	Weighted mode	9	0.025222	0.136435	0.857935
BMI_males	SnoringFemales	MR Egger	10	-5.60229	23.91778	0.820689
BMI_males	SnoringFemales	Weighted median	10	2.06383	0.965902	0.032623
BMI_males	SnoringFemales	Inverse variance weighted	10	3.356735	1.800137	0.062222
BMI_males	SnoringFemales	Weighted mode	10	1.784604	1.054656	0.124876
BloodPresure_males	SnoringFemales	MR Egger	10	21.98155	35.20387	0.549748
BloodPresure_males	SnoringFemales	Weighted median	10	3.948963	2.071496	0.056607
BloodPresure_males	SnoringFemales	Inverse variance weighted	10	2.529987	2.677373	0.344684
BloodPresure_males	SnoringFemales	Weighted mode	10	3.723509	2.936705	0.236644
PulseRate_males	SnoringFemales	MR Egger	10	27.12216	47.72998	0.585463
PulseRate_males	SnoringFemales	Weighted median	10	8.951877	3.928766	0.022694

PulseRate_males	SnoringFemales	Inverse variance weighted	10	6.289842	3.606065	0.081117
PulseRate_males	SnoringFemales	Weighted mode	10	9.08781	6.262307	0.180673
WholeBodyFat_males	SnoringFemales	MR Egger	10	-9.66392	40.9969	0.81957
WholeBodyFat_males	SnoringFemales	Weighted median	10	-0.22199	1.662024	0.893746
WholeBodyFat_males	SnoringFemales	Inverse variance weighted	10	3.395841	3.078202	0.269945
WholeBodyFat_males	SnoringFemales	Weighted mode	10	-0.74176	1.71222	0.675055
HeartAttack_males	SnoringFemales	MR Egger	10	0.978668	0.325255	0.016841
HeartAttack_males	SnoringFemales	Weighted median	10	0.033581	0.035821	0.348515
HeartAttack_males	SnoringFemales	Inverse variance weighted	10	0.07213	0.031556	0.022266
HeartAttack_males	SnoringFemales	Weighted mode	10	0.010273	0.050068	0.841988
WholeBodyFat_females	SnoringMales	GSMR	3	0.850404	1.97174	0.666252
BMI_females	SnoringMales	GSMR	3	-0.65158	1.01539	0.521063
PulseRate_females	SnoringMales	GSMR	3	4.51444	3.79559	0.234286
WholeBodyFat_males	SnoringFemales	GSMR	12	1.45647	1.01067	0.14956
BMI_males	SnoringFemales	GSMR	11	0.879139	0.541127	0.104238
PulseRate_males	SnoringFemales	GSMR	13	4.76402	2.44389	0.051252
SnoringMales	WholeBodyFat_females	GSMR	207	0.005064	0.00054	6.73E-21
SnoringMales	BMI_females	GSMR	211	0.010086	0.001029	1.16E-22
SnoringMales	PulseRate_females	GSMR	38	0.001035	0.000633	0.102234
SnoringFemales	WholeBodyFat_males	GSMR	144	0.005038	0.000598	3.53E-17
SnoringFemales	BMI_males	GSMR	159	0.013927	0.001101	1.09E-36
SnoringFemales	PulseRate_males	GSMR	23	-4.5E-05	0.000606	0.94027
SnoringFemales	HeartAttack_males	GSMR	11	-0.10442	0.082884	0.207742
SnoringMales	BloodPresure_females	GSMR	135	-0.00125	0.000644	0.051374

SnoringFemales	BloodPresure_males	GSMR	59	-0.00048	0.000781	0.539047
HeartAttack_females	SnoringMales	GSMR	3	0.000714	0.017554	0.967559
BloodPresure_females	SnoringMales	GSMR	3	0.047313	1.99967	0.981123
HeartAttack_males	SnoringFemales	GSMR	13	0.06311	0.022709	0.005451
BloodPresure_males	SnoringFemales	GSMR	13	2.5222	1.20421	0.036218

Supplementary Table 3. Sample sizes for discovery, sex-stratified and sensitivity GWAS analyses.

Model	Sample Size*	Snoring prevalence
Snoring	408,317	37.3%
Snoring Males	189,971	47.8%
Snoring Females	218,346	28.3%
BMI adjusted	407,066	37.3%
BMI adjusted Males	189,333	47.8%
BMI adjusted Females	217,733	28.3%

*Sample size for the GWAS using only individuals with non-missing phenotypic and genetic data and passing quality control ancestry filters (see methods)

Supplementary Table 4. Field codes and instances used from UK Biobank.

Description	Field Code	Instance
Age	21022	Single instance
BMI	21001	0
Current tobacco smoking	1239	0
Frequency of drinking alcohol	20414	Single instance
How are people in household related to participant	6141	0
Sex	31	Single instance
Townsend deprivation index	189	0
Average total household income before tax	738	0
Snoring	1210	0
