# Supplementary material for: Insights into the aetiology of snoring from observational and genetic investigations in the UK Biobank
Source: Nat Commun. 2020 Feb 14;11:817. doi: 10.1038/s41467-020-14625-1 (PMC7021827; doi:10.1038/s41467-020-14625-1)
Supplement: Supplementary file 5 — Reporting Summary [file 41467_2020_14625_MOESM5_ESM.pdf]

nature research

Corresponding author(s): Renteria ME & Cuellar-Partida G

Last updated by author(s): Jan 21, 2020

# Reporting Summary

Nature Research wishes to improve the reproducibility of the work that we publish. This form provides structure for consistency and transparency in reporting. For further information on Nature Research policies, seeAuthors & Referees and theEditorial Policy Checklist .

## Statistics

For all statistical analyses, confirm that the following items are present in the figure legend, table legend, main text, or Methods section.

| n/a | Confirmed | |
|---|---|---|
| ☐ | ☒ | The exact sample size (*n*) for each experimental group/condition, given as a discrete number and unit of measurement |
| ☐ | ☒ | A statement on whether measurements were taken from distinct samples or whether the same sample was measured repeatedly |
| ☐ | ☒ | The statistical test(s) used AND whether they are one- or two-sided<br>*Only common tests should be described solely by name; describe more complex techniques in the Methods section.* |
| ☐ | ☒ | A description of all covariates tested |
| ☐ | ☒ | A description of any assumptions or corrections, such as tests of normality and adjustment for multiple comparisons |
| ☐ | ☒ | A full description of the statistical parameters including central tendency (e.g. means) or other basic estimates (e.g. regression coefficient) AND variation (e.g. standard deviation) or associated estimates of uncertainty (e.g. confidence intervals) |
| ☐ | ☒ | For null hypothesis testing, the test statistic (e.g. *F*, *t*, *r*) with confidence intervals, effect sizes, degrees of freedom and *P* value noted<br>*Give P values as exact values whenever suitable.* |
| ☒ | ☐ | For Bayesian analysis, information on the choice of priors and Markov chain Monte Carlo settings |
| ☒ | ☐ | For hierarchical and complex designs, identification of the appropriate level for tests and full reporting of outcomes |
| ☐ | ☒ | Estimates of effect sizes (e.g. Cohen's *d*, Pearson's *r*), indicating how they were calculated |

*Our web collection on statistics for biologists contains articles on many of the points above.*

## Software and code

Policy information about availability of computer code

| | |
|---|---|
| Data collection | No particular software was used for data collection. |
| Data analysis | PLINK (1.9), MAGMA version LD Score regression (1.0.1), Complex Traits Genomics Virtual Lab (0.60a, http://www.genoma.io), BOLTLMM (2.3.2), GCTA (1.91.7), GSMR (1.0.8), MAGMA (1.07b) FUMA (online platform: https://fuma.ctglab.nl), Python (3.5), R (3.5.1). |

For manuscripts utilizing custom algorithms or software that are central to the research but not yet described in published literature, software must be made available to editors/reviewers. We strongly encourage code deposition in a community repository (e.g. GitHub). See the Nature Research guidelines for submitting code & software for further information.

## Data

Policy information about availability of data

All manuscripts must include a data availability statement. This statement should provide the following information, where applicable:
- Accession codes, unique identifiers, or web links for publicly available datasets
- A list of figures that have associated raw data
- A description of any restrictions on data availability

UK Biobank data was provided under application 25331. Summary statistics for the discovery GWAS will be deposited in the GWAS catalog database after publication.

# Field-specific reporting

Please select the one below that is the best fit for your research. If you are not sure, read the appropriate sections before making your selection.

# Life sciences study design

All studies must disclose on these points even when the disclosure is negative.

| | |
|---|---|
| Sample size | Data for all available individuals of European Ancestry on the UK-B with snoring data and passing genetic and phenotypic QC were included and this is reflected in the final sample size. |
| Data exclusions | Individuals of non-European ancestry were excluded to avoid population stratification bias. Participants who answered "I don't know" or "I would rather not answer" to the snoring item were also excluded from the analyses. |
| Replication | The AGDS was used as the 'target' sample for polygenic prediction using the summary statistics of the discovery sample (UK Biobank). However, due to the enormous sample size difference and lack of statistical power, no 2-sample replication was attempted in AGDS. To our knowledge, there is no other public or accessible large sample with snoring data to provide replication |
| Randomization | N/A |
| Blinding | N/A |

# Reporting for specific materials, systems and methods

We require information from authors about some types of materials, experimental systems and methods used in many studies. Here, indicate whether each material, system or method listed is relevant to your study. If you are not sure if a list item applies to your research, read the appropriate section before selecting a response.

## Materials & experimental systems

| n/a | Involved in the study | |
|---|---|---|
| ☒ | ☐ | Antibodies |
| ☒ | ☐ | Eukaryotic cell lines |
| ☒ | ☐ | Palaeontology |
| ☒ | ☐ | Animals and other organisms |
| ☐ | ☒ | Human research participants |
| ☒ | ☐ | Clinical data |

## Methods

| n/a | Involved in the study | |
|---|---|---|
| ☒ | ☐ | ChIP-seq |
| ☒ | ☐ | Flow cytometry |
| ☒ | ☐ | MRI-based neuroimaging |

## Human research participants

Policy information about studies involving human research participants

| | |
|---|---|
| Population characteristics | We used data of participants of the UK Biobank study (mean age 57.01, +/- 7.7) from the UK and the Australian Genetics of Depression Study (AGDS; mean age 45.5, +/- 16.3) comprised of Australian adults who had been prescribed antidepressants in the previous five years. Data on snoring was collected on both samples |
| Recruitment | UK Biobank recruited 500,000 people aged 40-69 between 2006 and 2010 at 22 recruitment centres aross the UK. AGDS recruitment was via a combination of public appeal (media campaign) and individual mailouts sent by Australia's Department of Human services on behalf of the chief investigators. |
| Ethics oversight | The UK Biobank study was approved bu the UK Biobank Ethics and Governance Council; the AGDS was approved by QIMR Berghofer Medical Research Intitiute's Human Research & Ethics Committee |

Note that full information on the approval of the study protocol must also be provided in the manuscript.
