# Supplementary material for: Insights into the aetiology of snoring from observational and genetic investigations in the UK Biobank
Source: Nat Commun. 2020 Feb 14;11:817. doi: 10.1038/s41467-020-14625-1 (PMC7021827; doi:10.1038/s41467-020-14625-1)
Supplement: Supplementary file 7 — Description of Additional Supplementary Files [file 41467_2020_14625_MOESM7_ESM.pdf]

Title: Supplementary Data 1.
Description: Genomic risk loci for discovery, sensitivity and sex-stratified analyses.

Title: Supplementary Data 2.
Description:FUMA output files for snoring and genetic correlations.

Title: Supplementary Data 3.
Description:FUMA output files for snoring adjusted for BMI and genetic correlations.
